# Biomechanics of humeral locking plate augmented with fibular strut allograft and intramedullary strut plate using finite element analysis

**DOI:** 10.1038/s41598-025-09848-5

**Published:** 2025-08-09

**Authors:** Cheng-Hung Lee, Li-Kun Hung, Yu-Chun Yen, Kuo-Chih Su

**Affiliations:** 1 Department of Orthopedics, Taichung Veterans General Hospital, Taichung, 407 Taiwan; 2 Department of Post-Baccalaureate Medicine, College of Medicine, National Chung Hsing University, Taichung, 402 Taiwan; 3 Chung Shan Hospital, Taipei City, 106 Taiwan; 4 Department of Medical Research, Taichung Veterans General Hospital, Taichung, 407 Taiwan; 5 Department of Medical Equipment Development and Application, HungKuang University, Taichung, 433 Taiwan

**Keywords:** Humeral locking plate, Proximal humeral fracture, Biomechanics, Finite element analysis, Biomedical engineering, Medical research

## Abstract

A humeral locking plate augmented with fibular strut allograft treated for proximal humeral fracture without internal structural support is satisfactory. While it is better clinically and biomechanically than the locking plate alone, it has disadvantages, including difficulty to obtain and the possibility of infection. Other alternative augmentation approaches are in demand. Therefore, the hypothesis of this study is whether intramedullary strut plate can replace fibular strut allograft as a surgical method while providing similar biomechanical performance. The finite element analysis (FEA) models were established based on three-dimensional computed tomography images. Computer-aided design implants were incorporated into the models. According to different implants, models were divided into four groups: the intact humerus, humeral locking plate alone (LP), humeral locking plate augmented with fibular strut allograft (FA), and humeral locking plate augmented with intramedullary strut plate (IMP). The displacements and von Mises stresses were measured on the models by simulating axial force, oblique force and torsion. Compared with the LP group, the displacements and von Mises stresses on the humerus and humeral locking plate in the FA, and IMP groups were all smaller in axial force, oblique force, and torsion. The biomechanical effects of FA and IMP in proximal humeral fracture without internal structural support were basically similar in terms of axial force, oblique force, and torsion. Findings provide useful new ideas for implant design. Our FEA simulation indicates that both the fibular strut allograft (FA) and intramedullary strut plate (IMP) offer similar biomechanical stability in treating proximal humeral fractures without internal structural support. This supports the hypothesis that the intramedullary strut plate can effectively replace the fibular strut allograft.

## Introduction

Proximal humeral fracture is a common trauma of the elderly, associated strongly with osteoporosis^1–3^. Open reduction and locking plate fixation are the preferred treatments^1–4^. However, osteoporotic or comminuted fractures may also lead to unfavorable consequences of loss of reduction or implant failure^4,5^. Medial column support is the cornerstone of fixation of proximal humeral fractures^6,7^. The cortical contact at the medial column is more accessible for 2-part fractures, but not for osteoporotic, medial comminuted, 3-part, and 4-part fractures. Combination with other augmentations is needed to achieve the support of the medial column. Such measures are bone void filler (fibular strut allograft, cancellous allograft or autograft, bone cement), inferomedial screws or calcar screws, and medial buttress plate^2,6,8–10^.

In comparing between the locking plate alone and augmented with fibular strut allograft, several researches pointed out that the fibular strut allograft improves clinical and radiological outcomes, thereby reducing complications. Bae et al.'s study with cadaveric humeri cyclical loads showed that the locking plate augmented with fibular strut allograft group has lesser displacements, markedly higher maximum failure loads, and stiffness measures compared to the locking plate alone group. Varus collapses, and plates bending are seen in the locking plate alone group, but no broken plates or screws in both groups^11^. Mathison et al. found that the locking plate fixation with fibular strut allograft significantly reduces relative movements at the interface under bending loads, by increasing the failure load of the constructs by 1.72 fold and increasing the construct’s initial stiffness by 3.84 fold^12^. Lee et al. indicated that when introducing the fibular strut allograft graft, the maximal load increases by >200%, regardless of using conventional or locking plates. The maximal load and stiffness of the locking plate with fibular strut allograft are both higher than the locking plate alone^13^. Dasari et al.'s meta-analysis reported a significant difference favoring augmentation with fibular strut allograft regarding the following: change in humeral head height (HHH) and neck-shaft angle (NSA), final American Shoulder and Elbow Surgeons (ASES) score, and lower risks in developing major complications^8^. Tang et al.'s meta-analysis study indicated that locking plate with fibular strut allograft is superior to locking plate fixation alone in terms of the changes in HHH and NSA, Constant-Murley score, the ASES score, visual analogue scale (VAS) score, the varus malunion rate, and the screw penetration rate. No intergroup difference exists in the rate of humeral head osteonecrosis^10^. Several biomechanical studies also present evidence of better biomechanical effects of the locking plate augmented with fibular strut allograft than the locking plate alone.

Despite the excellent clinical outcome of the fibula strut allograft, its downside includes difficult placement, and difficulty to obtain. The possibility of infection is also a concern^14,15^. In addition, the presence of intramedullary fibula complicates joint replacement surgery if required^16^. Developing an alternative method that can replace the intramedullary fibula would help overcome these limitations. Given these considerations, this study introduced the intramedullary strut plate (IMP) as a potential alternative. The IMP features an intramedullary plate design, aiming to simplify implantation, reduce infection risk, and provide enhanced structural support while maintaining comparable biomechanical stability to FA. Here, we aimed to evaluate the biomechanical properties of the intramedullary strut plate using Finite Element Analysis (FEA), which is widely utilized in orthopedic biomechanics research due to its ability to perform detailed stress, strain, and displacement analysis on complex structures such as bones and implants. FEA enables controlled and standardized testing conditions, reducing variability that may arise in cadaveric or in vitro experiments, ensuring a precise comparison of different fixation methods. Additionally, many previous biomechanical studies on the humerus have also employed FEA to evaluate implant stability and fixation strategies. Our main objective was to use FEA, performed with the commercial software ANSYS Workbench, to analyze the biomechanical effects of three implantation methods: the humeral locking plate alone, the humeral locking plate augmented with fibular strut allograft, and the humeral locking plate augmented with an intramedullary strut plate. ANSYS Workbench is a widely-used commercial software for FEA structural analysis. Additionally, it supports various material models and boundary conditions, enabling the simulation of structural behavior under different types of loads and environmental conditions. By analyzing the biomechanical stability of the IMP, this study aims to determine whether it can serve as a viable alternative to the fibular strut allograft, addressing its associated drawbacks while achieving comparable fixation performance. Thus, the hypothesis of this study is that the intramedullary strut plate can effectively replace the fibular strut allograft as a surgical method while providing similar biomechanical performance.

## Materials and methods

### Build a simulation geometry model

The proximal humerus model was established using the National Institutes of Health CT images (Visible Human Project) from a male cadaver. The finite element model used in this study was constructed from humerus CT images, which were obtained from the National Institutes of Health (NIH) Visible Human Project. The use of these medical images and the development of the computational model were reviewed and approved by the Institutional Review Board I & II of Taichung Veterans General Hospital. The IRB approval number is CE16108B, under the title "Using medical image (The Visible Human Project) provided by US NIH to build an analyzable model." We used the medical image circle selection software (Mimics Medical 21.0, Materialise, Leuven, Belgium) to circle the approximate humerus region in the CT images. The bone was segmented into cortical and cancellous regions. As for the humeral locking plate and screw parts, we used the computer-aided design (CAD) software (Solidworks 2016, Dassault Systemes SolidWorks Corp, Waltham, MA, USA) to establish all parts of the models. Finally, they were combined to include the humerus, fibula, plates and screws using the Boolean operation to generate computer models suitable for FEA. The generated three-dimensional computer models were finally imported into FEA software (ANSYS Workbench 18.2, ANSYS, Inc., Canonsburg, PA).

**Fig. 1:**
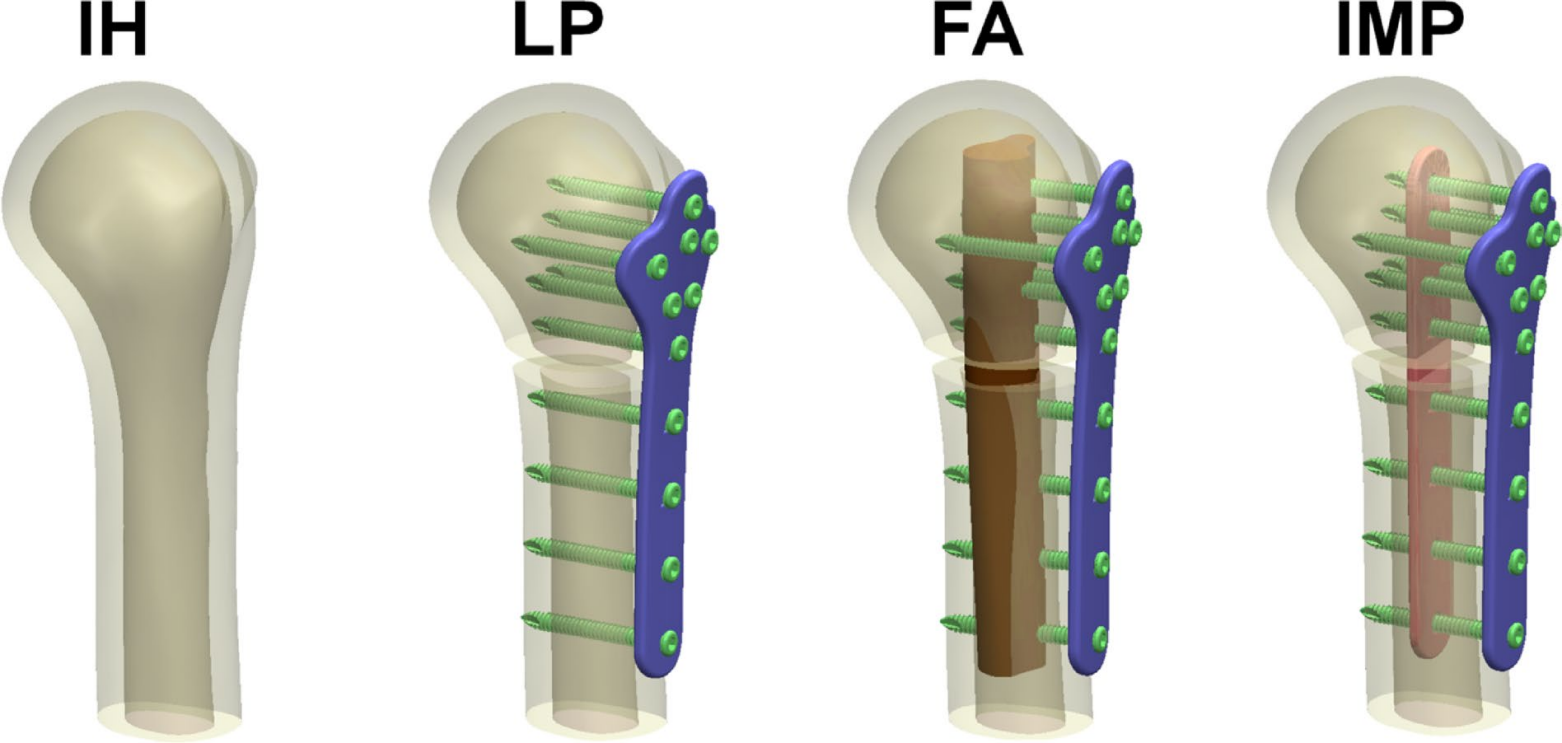
Four groups of computer FEA models, including intact humerus (IH), humeral locking plate alone (LP), humeral locking plate augmented with fibular strut allograft (FA), and humeral locking plate augmented with intramedullary strut plate (IMP).

**Fig. 2:**
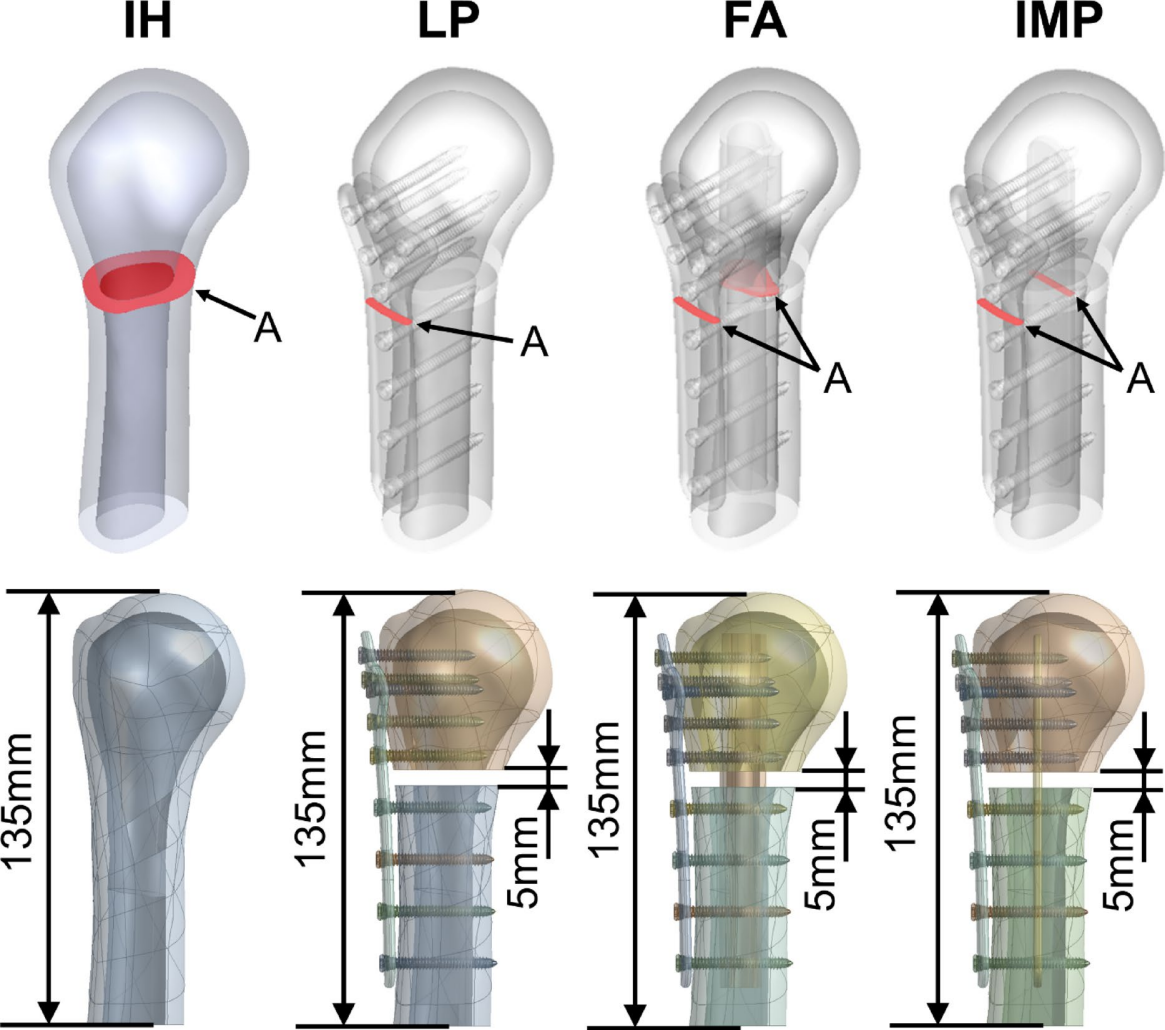
The cross-section of the fracture area (A, in red) and the height of the bone defect and the total length of the bone model.

In this study, four computer models were established, which were the intact humerus (IH), humeral locking plate alone (LP), humeral locking plate augmented with fibular strut allograft (FA) and humeral locking plate augmented with intramedullary strut plate (IMP) (Fig. 1). The height of the humerus used in this study is 135 mm. The dimensions of the humeral locking plate are 16–35 mm in length, 3 mm in width, and 120 mm in height. The screws used are 35 mm in length and 3 mm in diameter. The intramedullary strut plate design has dimensions of 16 mm in length, 2.5 mm in width, and 105 mm in height. The fibular strut allograft used has an approximate diameter of 10–15 mm and a height of 105 mm (The fibula computer model used in this study was also created from CT images provided by the Visible Human Project). The IMP used in this study is a non-locking plate, as it does not contain threads within its screw holes. However, the diameter of the holes on the intramedullary plate was designed to match the outer diameter of the locking screw threads, allowing the screws to fit snugly and engage with the plate upon insertion. This design ensures that the screws effectively secure the intramedullary plate within the construct. To simulate the biomechanical characteristics of a comminuted proximal humeral fracture, a 5 mm fracture gap was introduced at the fracture site in the LP, FA, and IMP models, thereby reducing direct structural support across the fracture. This approach represents a clinically relevant unstable fracture condition, suitable for evaluating the effectiveness of internal augmentation. Figure 2 shows the cross-section of the fracture area. Additionally, the cross-sectional area (A), moment of inertia (I), and polar moment of inertia (J) and their detailed information as shown in Table 1.

**Table 1:** The cross-sectional area, the moment of inertia, and the polar moment of inertia in the cross-section of the fracture area.

	IH	LP	FA	IMP
Cross-sectional area (A) (mm)	646.95	47.13	243.35	86.71
Moment of inertia (I) (mm^4^)	43,383.67	959.84	17,763.12	7949.07
Polar moment of inertia (J) (mm^4^)	69,234.00	996.81	23,123.64	9736.36

### Loading conditions and boundary conditions

**Fig. 3:**
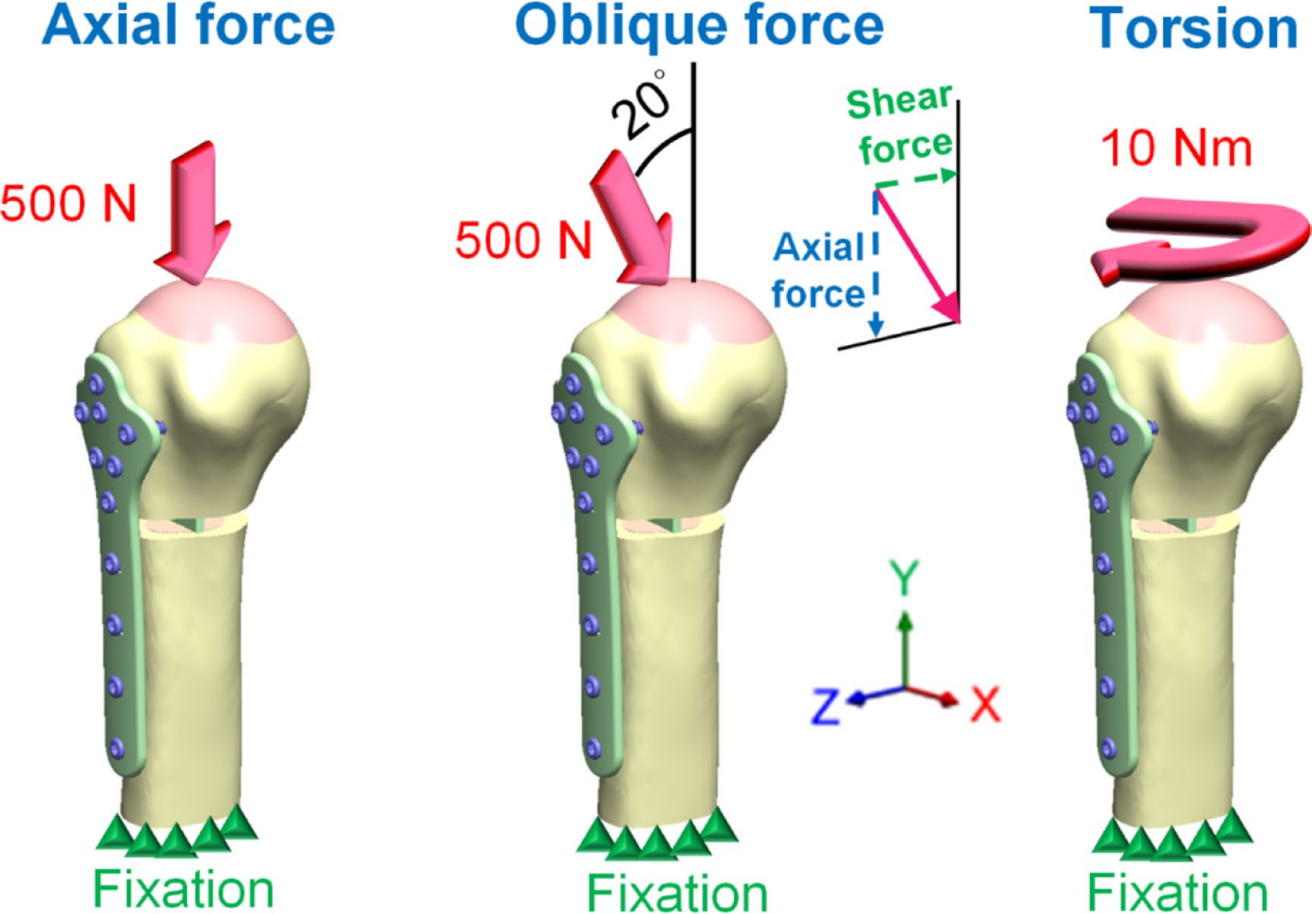
The boundary conditions and loading conditions in this study.

The boundary and loading conditions used for modelling, as shown in Fig. 3, followed those previous reports^17,18^. For the boundary conditions, the lower ends of the humeral shafts were fixed by the setting of displacement in the ANSYS Workbench software, indicated in Fig. 3 (green triangles), and the movements of the point on X-, Y-, and Z-axes were set as zero. The loading conditions were set as axial force, oblique force, and torsion. The axial force was defined as a downward force of 500 N acting at the top region of the humeral head (Fig. 3, pink area). The oblique force was defined as the humerus inclined at 20°, and a downward force of 500 N was applied to the top region of the humeral head, simulating a patient’s daily walking with crutches. The oblique force was divided into axial and shear forces. The torsion was defined as 10-Nm Torque applied to the top of the humeral head along the long axis of the humeral shaft.

### Material properties of the model

The computer FEA models had four materials: cortical bone, cancellous bone, plate, and screw. The material properties are shown in Table 2^17,19–22^. All materials were assumed to be homogeneous, isotropic, and linear elastic. Young’s modulus (E) and Poisson’s ratio (ν) were used to reflect the material properties. Plates and screws were simulated to be titanium alloys. The bone was also divided into the cortical and cancellous bones in both humerus and fibula. The interface settings between the IMP and bone, as well as the interface settings between the FA and bone, are both set to “bonded”.

**Table 2:** The material properties of this study^17,19–22^.

Material		Young’s modulus (MPa)	Poisson’s ratio
Humerus	Cortical bone	13,400	0.3
	Cancellous bone	2,000	0.3
Fibula	Cortical bone	17,000	0.3
	Cancellous bone	700	0.3
Plate		114,000	0.3
Screw		106,000	0.33

**Fig. 4:**
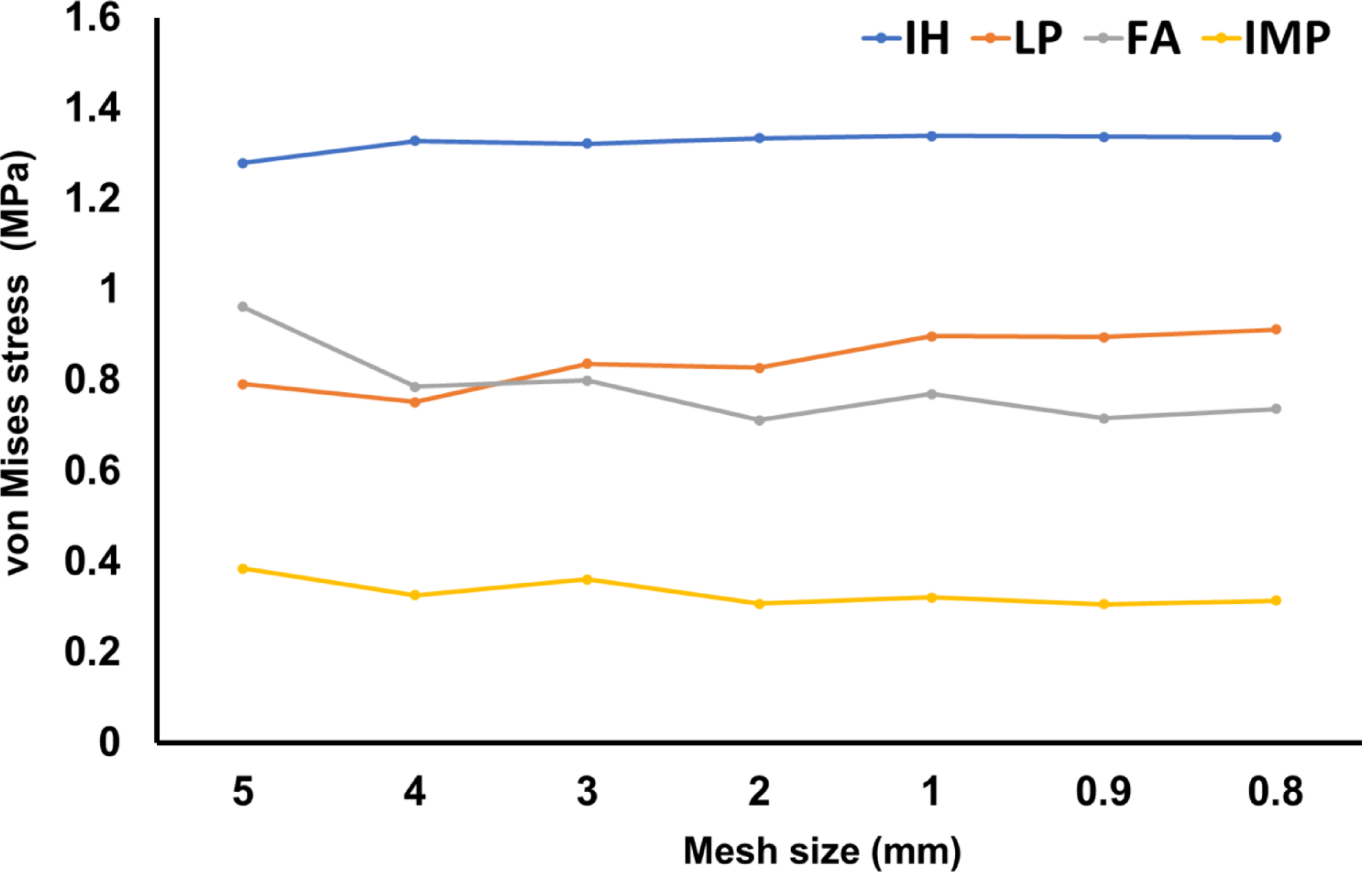
The results of the stress convergence tests at the same location on the humerus for the four different models in this study.

All four models passed the convergence test before starting the FEA to refine the results. The mesh size was adjusted to achieve convergence. Mesh elements were built on quadratic tetrahedral elements using ANSYS Workbench software. A downward force was applied to the humeral head as the loading condition, and the lower end of the humeral diaphysis was fixed as the boundary condition. Then, the stress value was used as the index of the numerical convergence observation to evaluate whether the model had reached the 5% convergence criterion. Figure 4 shows the results of the stress convergence tests at the same location on the humerus for the four different models in this study. The convergent results were that the mesh size was 0.9 mm, and Table 3 shows the number of nodes and elements of each group after meshing. Additionally, the finite element model was validated against a previous experimental study^23^, using the LP model as the reference. In that study, a 450 N load was applied, and the measured displacement ranged between 1.6 mm and 3.1 mm. In the finite element simulation, the LP model resulted in a displacement of 2.4713 mm, which falls within the experimental range, indicating that the finite element model is reasonable.

**Table 3:** The number of nodes and elements of each group after meshing.

Mesh number	IH	LP	FA	IMP
Number of nodes	150,423	1,632,537	1,657,660	1,665,198
Number of elements	87,271	954,990	954,467	970,858

After the FEA, the displacement and von Mises stress were used as the observation indices. The maximum displacement of the humerus was recorded, and so was the rotation angle to the y-axis of the pink region upon applied torsion (The rotation angle was obtained using ANSYS APDL (ANSYS Parametric Design Language) commands, specifically the ROTY(Measure_pilot) command, which calculates the rotation about the Y-axis.) (Fig. 3). The maximum displacement and rotation angle were used to calculate stiffness and evaluate the mechanics of the humerus under different structural supports. The von Mises stress was defined as

$$\sigma_{von} = \sqrt {\frac{1}{2}\left[ {\left( {\sigma_{1} - \sigma_{2} } \right)^{2} + \left( {\sigma_{1} - \sigma_{3} } \right)^{2} + \left( {\sigma_{2} - \sigma_{3} } \right)^{2} } \right]}$$

where ${\sigma }_{1}$, ${\sigma }_{2}$, and ${\sigma }_{3}$ represent the principal stress along the three coordinate axes. The stress distribution diagrams of the humerus, humeral locking plate, fibular strut allograft and intramedullary strut plate were recorded individually.

## Results

**Fig. 5:**
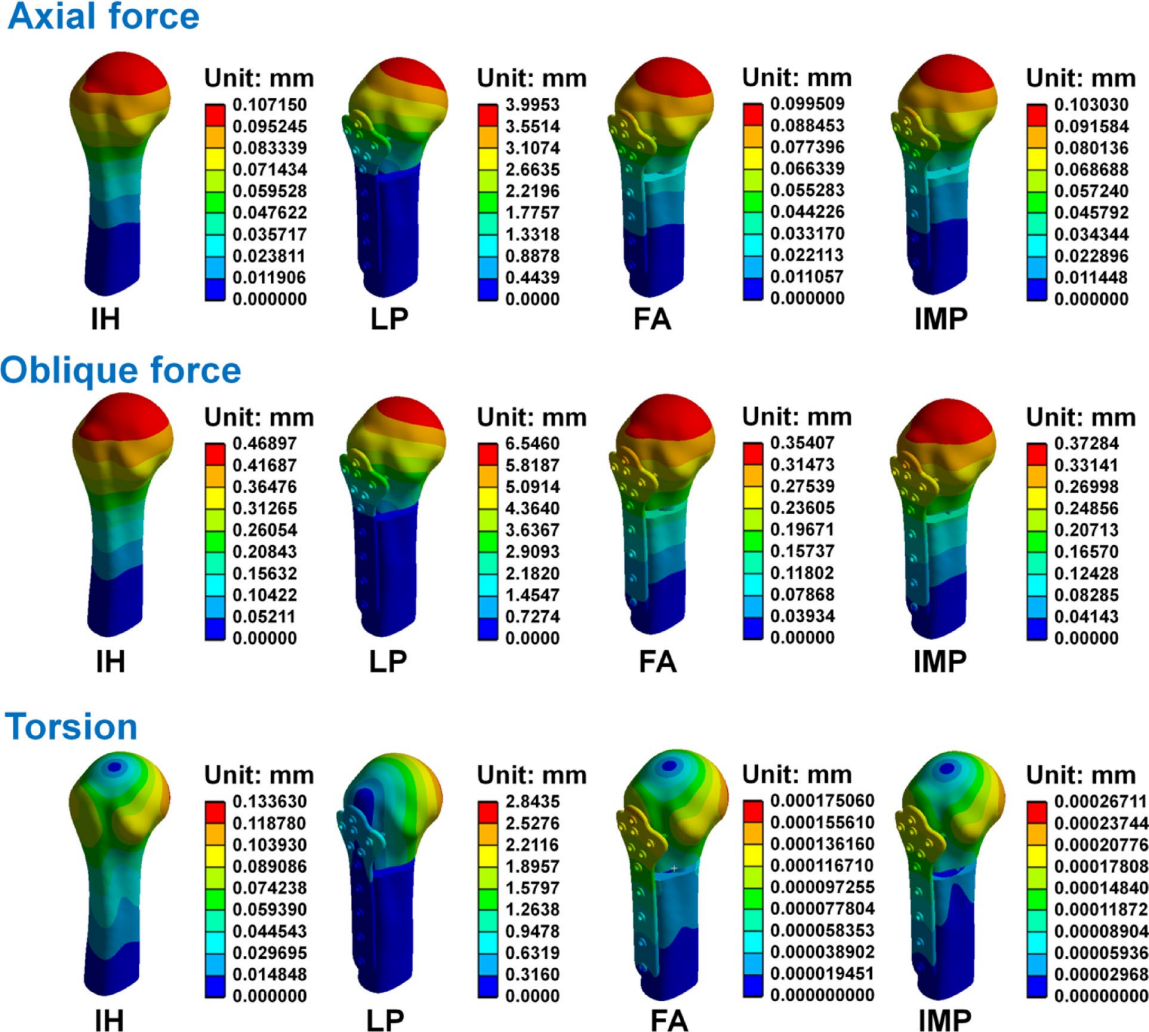
The displacement distributions of the humerus and different internal fixators under axial force, oblique force, and torsion.

The maximum displacement, rotation angle and stiffness on the humerus with different internal fixators under axial force, oblique force, and torsion are shown in Table 4. Displacement distributions of the humerus and different internal fixators under axial force, oblique force, and torsion are shown in Fig. 5. Results revealed that when these models were under three such loading conditions, the largest displacement was found in the LP group. In comparison, displacement of the FA and IMP groups were rather similar, with the FA displacement being slightly smaller. In each group, the location of the maximum displacement occurs near the top region of the humeral head. The stiffness was calculated to reflect the strengths of structures. Under external loading conditions of axial force, oblique force, and torsion, the FA group exhibits higher stiffness, with values of 5024.6711 N/mm, 1412.1501 N/mm, and 33110.3900 N/°, respectively. Higher stiffness in a structure indicates that a larger external force is required to produce greater displacement and rotation angles, suggesting that the structure has better stability. The construct stiffness is one measure of the overall stability of the entire system. A fixation with better structural stability facilitates fracture healing, allowing patients to begin postoperative movement sooner to restore shoulder joint function^17^.

**Table 4:** The maximal displacement, rotation angle and stiffness on the humerus under axial force, oblique force, and torsion.

	Axial force				Oblique force				Torsion			
	IH	LP	FA	IMP	IH	LP	FA	IMP	IH	LP	FA	IMP
Maximal displacement (mm)	0.1072	3.9953	0.0995	0.1030	0.4690	6.5460	0.3541	0.3728	0.1336	2.8435	0.0002	0.0003
Rotation angle (°)	–	–	–	–	–	–	–	–	0.2393	3.1929	0.0003	0.0004
Stiffness (N/mm)	4666.3556	125.1470	5024.6711	4852.9554	1066.1663	76.3825	1412.1501	1341.0578	–	–	–	–
Stiffness(N/°)	–	–	–	–	–	–	–	–	41.7903	3.1319	33,110.3900	22,510.8615

**Fig. 6:**
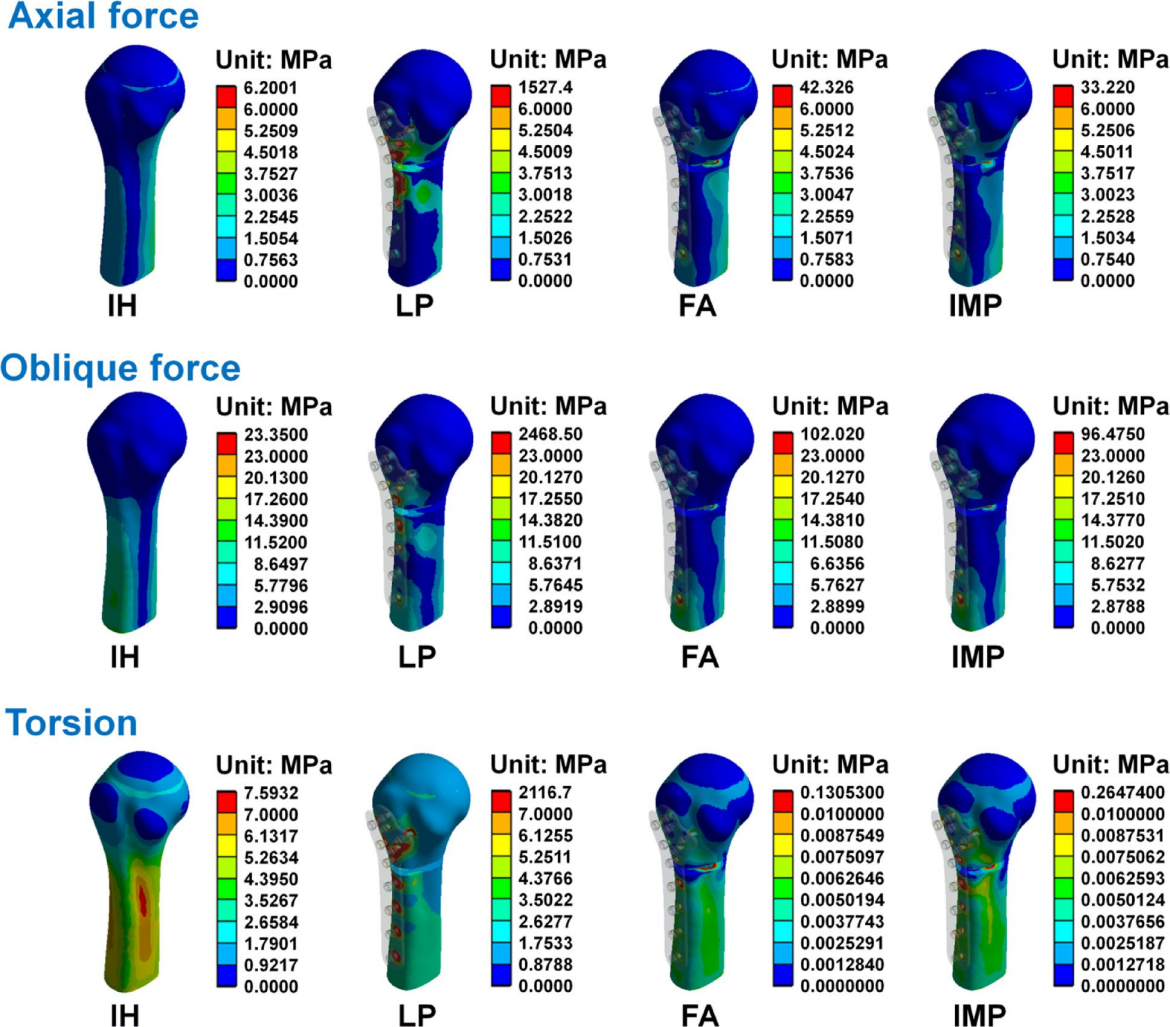
The von Mises stress distributions of the humerus in each group under the axial force, oblique force, and torsion.

**Fig. 7:**
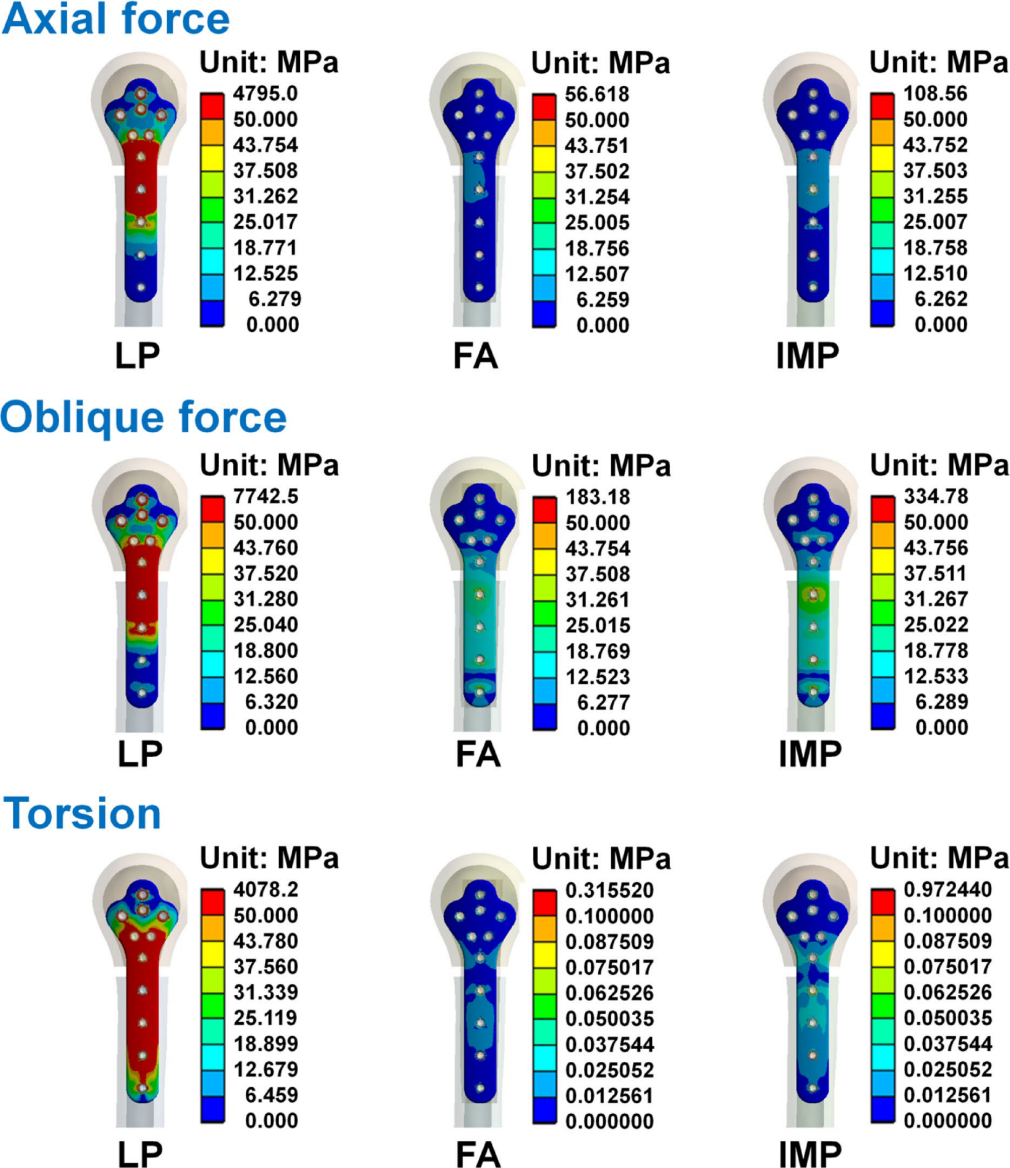
The von Mises stress distributions of the humeral locking plate in each group under the axial force, oblique force, and torsion.

**Fig. 8:**
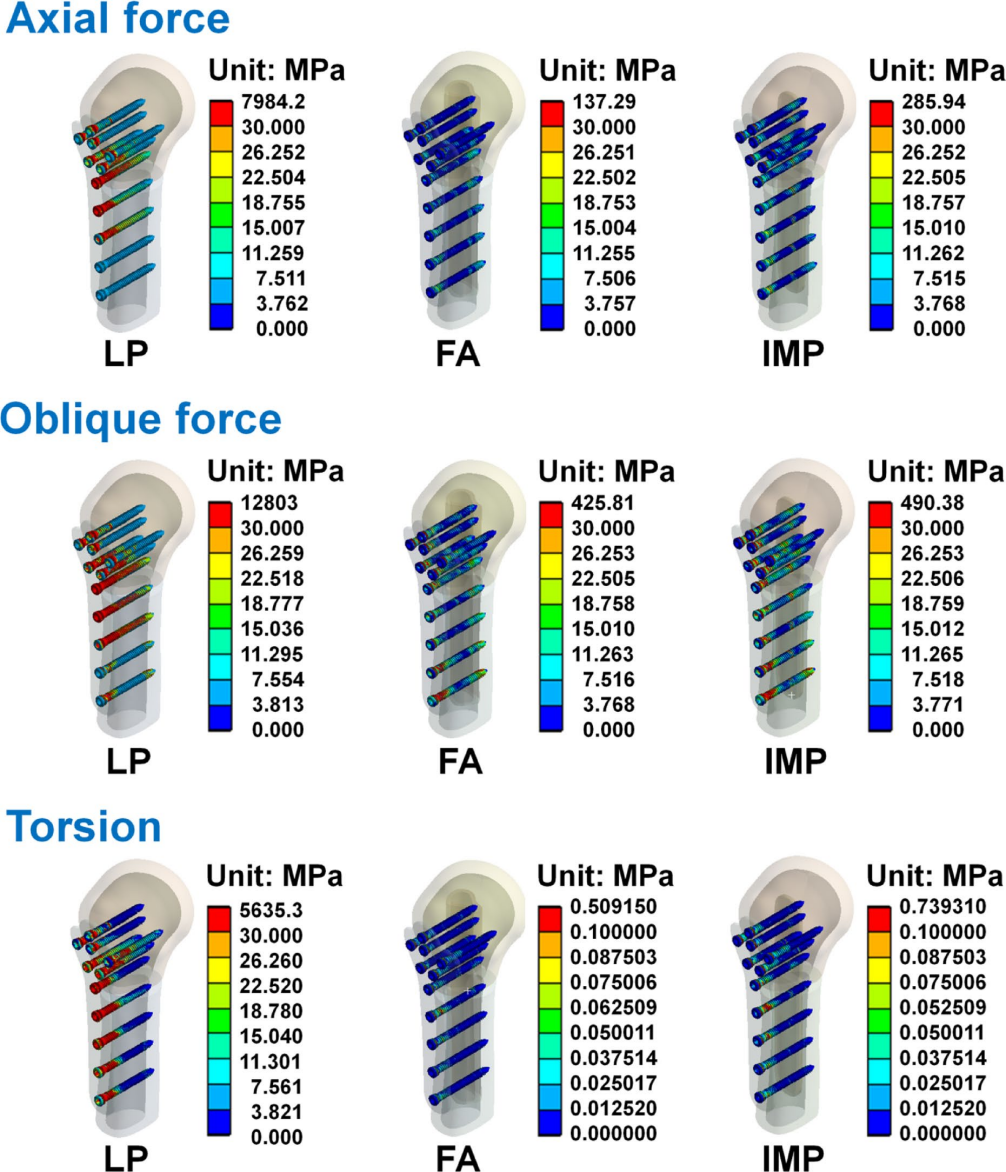
The von Mises stress distributions of the screws in each group under the axial force, oblique force, and torsion.

**Fig. 9:**
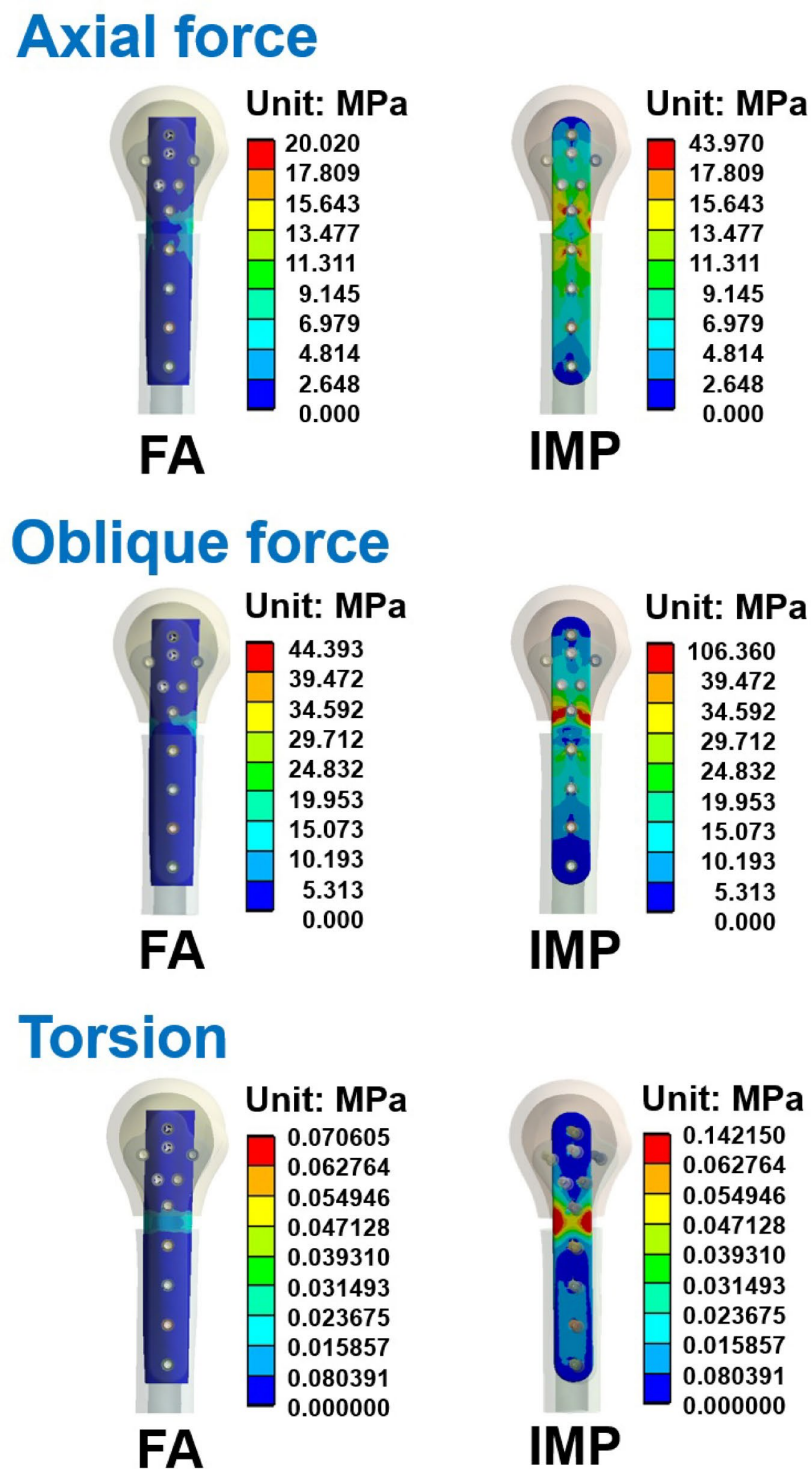
The von Mises stress distributions of the fibular strut allograft and intramedullary strut plate in the FA and IMP groups under the axial force, oblique force, and torsion.

The von Mises stress distribution of the humerus, humeral locking plate, and screws in each group are shown in Figs. 6, 7 and 8. The maximum von Mises stress in each group is shown in Table 5. Results showed that when the humerus models were subjected to the three different loading conditions, the humerus, humeral locking plate, and screws in the LP group had larger von Mises stress. Whereas the von Mises stresses were similar between FA and IMP groups, with the LP group being slightly smaller. Figure 7 shows the stress on the humeral locking plate in the LP, FA, and IMP groups. It was observed that the humeral locking plate in the LP group has higher stress near the humeral defect area compared to the other two groups. In structural analysis, when an object experiences external forces that generate high stress, exceeding its yield strength can result in failure. Thus, after the humeral locking plate is implanted in the humerus, it is essential to minimize high stress to prevent the plate from fracturing. The von Mises stress distributions of the fibular strut allograft and intramedullary strut plate under axial, oblique, and torsional loading conditions are shown in Fig. 9. High stress concentrations were observed near the fracture site in both the FA and IMP groups across all loading modes. Among them, the intramedullary strut plate exhibited higher stress levels than the fibular strut allograft. Although Table 5 shows that the LP group exhibited relatively high von Mises stress values, further analysis revealed that the peak stresses were primarily concentrated at the thread tips of the screws. In finite element analysis, models that include detailed thread geometry often produce localized stress concentrations at the threads, resulting in elevated stress values in these regions^24,25^. To avoid the influence of such thread induced stress artifacts and to ensure consistent visual comparisons across groups, we manually adjusted the second color level in the von Mises stress nephograms to remain below the yield strength of bone and titanium alloy, using the same value wherever possible. This standardization allowed for a more objective assessment of stress distribution differences among the groups. Based on the stress nephograms, the LP group clearly exhibited a broader area of high stress compared to the FA and IMP groups.

**Table 5:** The maximal von Mises stress on the humerus, humeral locking plate, fibular strut allograft and intramedullary strut plate.

	Axial force				Oblique force				Torsion			
	IH	LP	FA	IMP	IH	LP	FA	IMP	IH	LP	FA	IMP
Humeral cortical bone (MPa)	6.200	1527.400	42.326	33.220	23.350	2468.500	102.020	96.475	7.593	2116.700	0.131	0.265
Humeral cancellous bone (MPa)	0.456	199.940	7.870	13.071	1.483	322.770	12.196	21.387	0.672	192.830	0.020	0.042
Humeral locking plate (MPa)	–	4795.000	56.618	108.560	–	7742.500	183.180	334.780	–	4078.200	0.316	0.972
Screws (MPa)	–	7984.200	137.290	285.940	–	12,803.000	425.810	490.380	–	5635.300	0.509	0.739
Fibular strut cortical bone (MPa)	–	–	20.020	–	–	–	44.393	–	–	–	0.071	–
Fibular strut cancellous bone (MPa)	–	–	2.392	–	–	–	6.947	–	–	–	0.006	–
Intramedullary strut plate (MPa)	–	–	–	43.970	–	–	–	106.360	–	–	–	0.142

## Discussion

This study evaluated biomechanical analysis on humeral locking plate alone, augmented with fibular strut allograft and intramedullary strut plate in proximal humeral fractures. The outcomes of this study were the displacement and von Mises stress value of the structures. The displacement and rotation angles of the LP group were both larger than those of the FA and IMP groups, regardless of axial force, oblique force, and torsion. About von Mises stress, regardless of axial force, oblique force, and torsion, both the FA and IMP groups showed lower stress at the humeral cortical bone, cancellous bone, humeral locking plate and screws compared to the LP group. Therefore, the presence of intramedullary struts can enhance overall biomechanical stability. The results of the study show that the use of IMP had similar biomechanical stability to FA, which is consistent with the hypothesis of this study. Our findings align with those of previous study^23^, which have demonstrated that the addition of FA enhances the stability of humeral fracture fixation when combined with a locking plate. Prior research has shown that FA reduces displacement and improves fixation stiffness, which is consistent with our FEA results. In our study, the FA group exhibited significantly improved stability compared to the LP group across all loading conditions, further supporting the role of FA in enhancing structural integrity. Furthermore, a recent study has also investigated the use of an intramedullary plate for proximal humeral fracture fixation, demonstrating its potential in enhancing biomechanical stability^26^. However, there are notable differences between that study and the present research. The previous study did not include a direct comparison with the locking plate (LP) group, which limits its ability to assess the relative effectiveness of intramedullary plate fixation against standard plating techniques. In contrast, our study incorporates a comprehensive comparison among LP, FA, and IMP groups, providing a more complete biomechanical evaluation. Additionally, in our study, the intramedullary plate was designed to be secured to the lateral locking plate using seven screws, ensuring stronger interlocking fixation. The length of the intramedullary plate used in our model is also designed to match that of the fibular strut allograft, whereas the previous study utilized an intramedullary plate of a different length. These design differences may influence biomechanical performance and fixation stability.

**Fig. 10:**
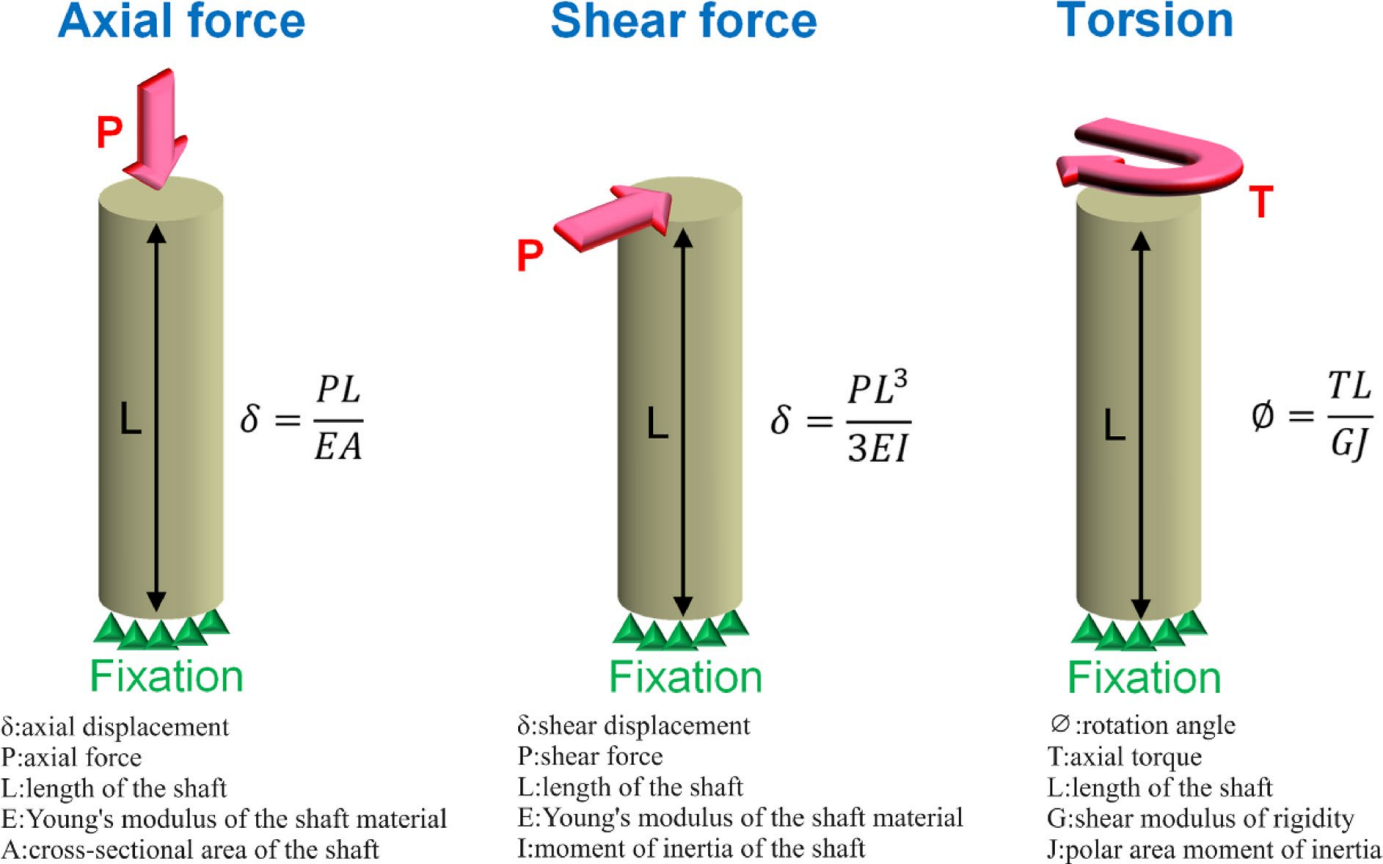
The displacements and rotation angles are subject to axial force, oblique force, and torsion. The oblique force can be resolved into an axial force and a shear force.

Given a 500 N axial force as the loading condition, the displacements of various groups were observed. The LP group had the most significant displacement compared to other groups. According to material mechanics, when an axial structure is subjected to an axial force, as shown in Fig. 10, its displacement can be expressed by the formula δ=PL/EA, where δ is the axial displacement, P is the axial force, L is the length of the shaft, E is Young’s modulus of the shaft material, and A is the cross-sectional area of the shaft. When the axial force to the shaft and the length of the shaft are both kept constant, the shaft displacement is inversely proportional to Young’s modulus and the cross-sectional area of the shaft. Therefore, considering only structural characteristics, the smaller the cross-sectional area of the structure, the greater is the displacement. Thus, according to the cross-sectional areas of the fracture area of various groups, as shown in Table 1, one can deduce that since the LP group has a smaller cross-sectional area, it generates more significant deformation. Additionally, it was observed that although the cross-sectional area of the IH group is larger than that of the other three groups, its displacement is not the smallest. This is primarily because, under axial force, factors influencing structural displacement include not only the cross-sectional area but also the Young’s modulus. Since the model used in this study is not a single-material model, it is challenging to evaluate it using a single material’s Young’s modulus. However, the IH group did not use metal materials, resulting in a relatively smaller average Young’s modulus for IH group. This is one of the reasons why, despite having a larger cross-sectional area, the IH group does not exhibit the smallest displacement.

When a 500 N force was applied to the humerus at an inclined angle of 20°, i.e., as an oblique force which can be resolved into an axial force and a shear force (Fig. 3), as the loading condition, the LP group produced the most significant displacement. According to material mechanics, when an axial structure is subjected to a shear force, as shown in Fig. 10, its displacement is determined by the formula δ = (PL^3^)/3EI, where δ is the axial displacement, P is the shear force, L is the length of the shaft, E is Young’s modulus of the shaft material, I is the moment of inertia of the shaft. Therefore, when the shear force to the shaft and the length of the shaft are kept constant, the deformation of the shaft is inversely proportional to Young’s modulus and the moment of inertia of the shaft. Therefore, considering only structural characteristics, the smaller the moment of inertia of the structure, the greater is the displacement. Thus, from the moment of inertia in the fracture area of various groups, as shown in Table 1, one can deduce that since the LP group has a smaller moment of inertia, it produces more significant deformation. Additionally, although the moment of inertia of the shaft in the IH group is greater than in the other three groups, its shear displacement is not the smallest. This is primarily because, under shear force, factors influencing structural displacement include not only the moment of inertia but also the Young’s modulus. Due to the complexity of the model used in this study, it is difficult to assess it using a single material’s Young’s modulus. Moreover, since the IH group did not use metal materials, its average Young’s modulus is relatively lower. Therefore, the IH group does not exhibit the smallest shear displacement.

When the torsion of 10 Nm was given as the loading condition, the LP group showed the greatest rotational displacement compared with other groups. According to material mechanics, when an axial structure is subjected to torsion, as shown in Fig. 10, its rotation angle can be calculated by the formula ∅ = TL/GJ, where ∅ is the rotation angle, T is the axial torque, L is the length of the shaft, G is the shear modulus of rigidity (G = E/2(1 + ν), G is related mostly to Young’s modulus and Poison’s ratio), and J is the polar area moment of inertia. Therefore, when the torque to the shaft and the length of the shaft are kept constant, the rotation angle of the shaft is inversely proportional to the shear modulus of rigidity and the polar area moment of inertia of the shaft. If considering only the structural characteristics, the smaller the polar area moment of inertia of the structure, the greater is the rotation angle. Thus, from the polar area moment of inertia at the fracture area of various groups, as shown in Table 1, one can deduce that since the LP group has a smaller polar area moment of inertia, it generates a larger rotation angle. Additionally, although the polar moment of inertia in the IH group is greater than that in the other three groups, its rotation angle is not the smallest. This is primarily because, under axial torque, factors influencing the rotation angle of the structure include not only the polar moment of inertia but also the shear modulus of rigidity. The shear modulus of rigidity is mainly proportional to Young’s modulus (since the Poisson’s ratio values for the various materials used in this study are similar). Moreover, due to the complexity of the model used in this study, it is difficult to assess it using a single material’s Young’s modulus. Since the IH group did not use metal materials, its average shear modulus of rigidity is relatively lower. This is one of the reasons why the IH group does not exhibit the smallest rotation angle.

Comparing between the FA and IMP groups, they had similar displacement and rotation angles regardless of the axial force, oblique force, and torsion. The structure simulated in this study was not simple but represented composites of various materials. It was difficult to determine Young’s modulus and shear modulus of rigidity on the fracture surface. However, fibular strut allograft can increase the thickness of the cortex in the shaft, thus increasing the stiffness of the overall structure. Likewise, the intramedullary strut plate had metal support in the shaft, which also increased the stiffness of the overall structure. According to our FEA results, as shown in Table 4, the FA and IMP groups had better stability compared to the LP group, with approximately similar displacement and rotation angle. Although IMF replaces fibular strut allograft with an intramedullary strut plate, in this study, both are positioned centrally on the humerus, providing enhanced support for humeral fractures. Therefore, when comparing the LP, FA, and IMP groups, the FA and IMP groups exhibit more similar biomechanical behavior.

Observing the stress on the humeral locking plate in the LP, FA, and IMP groups, the LP group exhibits higher stress on the humeral locking plate. This is primarily because, under axial force, oblique force, and torsion, the LP group relies solely on the humeral locking plate for support at the fracture site, resulting in greater deformation of the plate. According to Hooke’s Law, σ = E∈, where σ is stress, E is Young’s modulus, and ∈ is strain, stress is proportional to strain, and deformation is related to strain. Therefore, stress and deformation are positively correlated. Consequently, the humeral locking plate in the LP group shows more pronounced high stress. Additionally, previous studies have indicated that the fracture of the plate at the site of humeral neck fractures may also be caused by cyclic loading. Exceeding the fatigue limit of titanium alloys with cyclic loading can result in unavoidable fractures of the plate following surgery^22^. Under high-stress conditions, cyclic loading may result in larger stress amplitudes, significantly reducing the fatigue life of the material and leading to fatigue failure.

Both the FA and IMP groups had intramedullary struts acting as internal structural support. Regardless of a fibular strut allograft or an intramedullary strut plate, the result was reduced stress at the humeral locking plate and screws, hence avoiding implant breakage due to metal fatigue. In addition, stress concentration was observed on both the fibular strut allograft and the intramedullary strut plate near the fracture site under all loading conditions, as shown in Fig. 9. The intramedullary strut plate exhibited relatively higher von Mises stress than the fibular strut allograft, which is primarily due to its higher Young’s Modulus. The LP group had no internal structural support, and the force was transmitted only through the humeral locking plate. There was high stress on the humeral locking plate, leading to a higher risk of implant breakage. Micic et al. reported complications of humeral plate breakage four weeks after surgery^5^. Possible risk factors include malreduction, loss of medial support, and negligence of tension band sutures on the tuberosities. Kim et al. pointed out that varus collapse has poor clinical results, and the humeral locking plate with fibular strut allograft has better radiologic and clinical outcomes^9^. Therefore, the use of the fibular strut allograft or intramedullary strut plate for plate fixation is a potential treatment option for patients with internal column instability.

There are some limitations of our study. This study created a bone defect at the humeral neck to simulate proximal humeral fracture without any medial support. However, the condition of clinical proximal humerus fractures could vary widely. There may be several bone fragments and soft tissue in the internal column of the fracture. In theory, it can provide a small part of support, without complete bone defect. If the bone defect is filled with a cancellous bone graft, similar clinical results to FA augmentation treatment may be obtained^23^. However, to demonstrate the effects of different implants on humeral fracture fixation in the biomechanical analysis, this study created the fracture model in a manner similar to previous studies^17,26^. In addition, the material properties were assumed to be homogeneous, isotropic, and linearly elastic, in line with previous studies. Although these hypothetical results were slightly different from the actual situation, the trend of the results of the different implants likely holds, with a better understanding of the impacts of the parameters. In clinical practice, the calcar screw is commonly used as an augmented treatment for medial support, but the humeral locking plate in this study did not adopt the design of the calcar screw. According to the clinical study of Kim et al., who compared the clinical and radiographic results of fibular strut allograft and calcar screw in the proximal humeral 3-part and 4-part fracture, fibular strut allograft in 4-part fracture has better clinical outcomes, whereas 3-part and 4-part fractures have better radiographic results and fewer complications^25^. Therefore, fibular strut allograft can achieve a better curative effect than the calcar screw for more comminuted fractures, in the absence of internal structural support. Therefore, to simplify the variables, this study design was only for the mechanical analysis of the fibular strut allograft, without incorporating in the bone plate a calcar screw. A uniform screw orientation (parallel configuration) was adopted across all models to minimize confounding variables, as angled screw placement through the intramedullary plate is technically challenging and would make precise alignment within the narrow humeral canal difficult. Additionally, to avoid any impact of using screws of different lengths on the results, this study utilized screws of the same length as the model. Furthermore, the selection of a specific humeral locking plate in this study was based on the need to securely fix the FA or IMP within the construct. Many commercially available humeral locking plates have limited screw hole configurations in the middle region, and in some designs, the holes are asymmetrical or absent in the central portion of the plate, which may limit their ability to achieve optimal fixation when combined with FA or IMP. Therefore, a customized plate with centrally positioned screw holes was used in this study to allow for the proper integration of FA and IMP. This specific design ensured that the plate could effectively secure the fibular strut allograft and intramedullary strut plate, making their combination with the humeral locking plate feasible for biomechanical evaluation. The IMP used in this study is a non-locking plate, as it does not contain threads within its screw holes. However, the diameter of the holes on the intramedullary plate was designed to match the outer diameter of the locking screw threads, allowing the screws to fit snugly and engage with the plate upon insertion. This design ensures that the screws effectively secure the intramedullary plate within the construct. Additionally, muscle structures, such as the rotator cuff, were not included in the finite element analysis model. The soft tissues surrounding the proximal humerus play a role in joint stability and load transmission, which may introduce additional biomechanical effects. However, due to the complexity of soft tissue modeling and its interactions with implants, this study focused solely on the bone and fixation constructs (IH, LP, FA, IMP) to ensure a controlled and simplified comparison of fixation stability. Although this simplification differs from real physiological conditions, the overall trend of the simulation results should remain valid for evaluating the relative stability of different fixation methods. Additionally, in the design of the lateral plate model, the highest point of the plate is typically positioned 5–8 mm below the apex of the greater tuberosity in clinical practice. However, this study primarily aimed to evaluate whether the IMP could serve as an alternative to the FA in terms of biomechanical stability. Therefore, these factors were not specifically considered in the design of this study. In this study, the Young’s Modulus of cortical bone was set at 13,400 MPa, representing that of a normal individual. While bone quality, including osteoporosis, can affect fixation stability, incorporating such factors introduces additional variables beyond the scope of this study. Therefore, we used material properties representing normal cortical bone to ensure a clear and direct comparison of FA and IMP under standardized conditions. The effect of osteoporosis on different fixation methods could be an important topic for future research.

This study primarily utilizes FEA to investigate the biomechanical effects following the implantation of the humeral locking plate alone, augmented with fibular strut allograft, and augmented with intramedullary strut plate. In the future, further discussion and analysis can be conducted on additional factors, such as the addition of a proximal humerus nail for comparison, alterations in the diameter, width, or canal filling percentage of the intramedullary plate, and comparisons involving screws penetrating or not penetrating the intramedullary plate. These explorations are likely to provide valuable insights for the future design of innovative humerus plates and address the limitations associated with fibular strut allograft implantation.

## Conclusions

The displacement and stress of the structure in the FA and IMP groups are much smaller than in the LP group, and the biomechanical behaviors between the FA and IMP groups are similar. We concluded that in the proximal humeral fracture without internal structural support, the biomechanical stability and stress analysis of using proximal humeral locking plate augmented with fibular strut allograft or intramedullary strut plate is better than that of proximal humeral locking plate alone, and, encouragingly, the two have approximately equivalent mechanical performance. Compared to FA, IMP offers advantages such as eliminating graft availability concerns, reducing donor-site complications, and simplifying surgical procedures, making it a promising alternative for clinical applications. However, while this study suggests that IMP can serve as a substitute for FA, further clinical validation through cadaveric or in vivo studies is necessary before drawing firm conclusions. As this study is based on FEA, additional experimental and clinical investigations are required to confirm its real-world effectiveness. Despite these limitations, the findings provide valuable biomechanical insights and rationale for considering intramedullary strut plates as an alternative to fibular strut allografts in proximal humeral fracture fixation.

## Data Availability

The data used and/or analyzed in the current study are available from the corresponding author upon reasonable request.
